# Clinical, Imaging and Neurogenetic Features of Patients with Gliomatosis Cerebri Referred to a Tertiary Neuro-Oncology Centre

**DOI:** 10.3390/jpm13020222

**Published:** 2023-01-27

**Authors:** David Doig, Lewis Thorne, Jeremy Rees, Naomi Fersht, Michael Kosmin, Sebastian Brandner, Hans Rolf Jäger, Stefanie Thust

**Affiliations:** 1 Lysholm Department of Neuroradiology, National Hospital for Neurology and Neurosurgery, Queen Square, London WC1N 3BG, UK; 2 Victor Horsley Department of Neurosurgery, National Hospital for Neurology and Neurosurgery, Queen Square, London WC1N 3BG, UK; 3 Department of Neurology, National Hospital for Neurology and Neurosurgery, Queen Square, London WC1N 3BG, UK; 4 Department of Neuro-Oncology, National Hospital for Neurology and Neurosurgery, Queen Square, London WC1N 3BG, UK; 5 Department of Neurodegenerative Disease, UCL Institute of Neurology and Division of Neuropathology, National Hospital for Neurology and Neurosurgery, London WC1N 3BG, UK; 6 Neuroradiological Academic Unit, Department of Brain Rehabilitation and Repair, UCL Institute of Neurology, Queen Square, London WC1N 3BG, UK; 7 Imaging Department, University College Hospital, London WC1N 3BG, UK

**Keywords:** glioma, astrocytoma, glioblastoma, neuroimaging, magnetic resonance imaging, neuropathology

## Abstract

Introduction: Gliomatosis cerebri describes a rare growth pattern of diffusely infiltrating glioma. The treatment options are limited and clinical outcomes remain poor. To characterise this population of patients, we examined referrals to a specialist brain tumour centre. Methods: We analysed demographic data, presenting symptoms, imaging, histology and genetics, and survival in individuals referred to a multidisciplinary team meeting over a 10-year period. Results: In total, 29 patients fulfilled the inclusion criteria with a median age of 64 years. The most common presenting symptoms were neuropsychiatric (31%), seizure (24%) or headache (21%). Of 20 patients with molecular data, 15 had IDH wild-type glioblastoma, with an IDH1 mutation most common in the remainder (5/20). The median length of survival from MDT referral to death was 48 weeks (IQR 23 to 70 weeks). Contrast enhancement patterns varied between and within tumours. In eight patients who had DSC perfusion studies, five (63%) had a measurable region of increased tumour perfusion with rCBV values ranging from 2.8 to 5.7. A minority of patients underwent MR spectroscopy with 2/3 (66.6%) false-negative results. Conclusions: Gliomatosis imaging, histological and genetic findings are heterogeneous. Advanced imaging, including MR perfusion, could identify biopsy targets. Negative MR spectroscopy does not exclude the diagnosis of glioma.

## 1. Introduction

The term “gliomatosis cerebri” describes the diffuse infiltration of glioma cells through three or more lobes of the brain [1,2]. Previously a distinct diagnosis, since the publication of the 2016 and 2021 World Health Organization Classification of Tumours of the Central Nervous System, gliomatosis is no longer recognised as a specific neoplasm, but considered a histological pattern comprising different molecular classes of glioma. MRI remains the best imaging modality to characterise brain tumours, but may not detect the full extent of glioma microinfiltration.

The treatment of gliomatosis is limited to radiotherapy and/or chemotherapy, and the role of surgery is confined to biopsy alone due to the diffuse infiltrative nature of the disease. The benefit of radiotherapy is limited by the extent of brain involvement and the large radiation field that is required, and no randomised trial has demonstrated any particular chemotherapy combination to be effective in terms of tumour response or progression-free survival [3] (although patients receiving chemotherapy in general tend to have longer survival than those who do not [4]). Five-year survival rates are correspondingly poor at under 20% [5].

The aim of this retrospective study was to summarise the clinical, imaging, and histological characteristics of patients with gliomatosis cerebri referred to our institution, with a view to identifying opportunities for improvement of the diagnostic pathway.

## 2. Material and Methods

### 2.1. Inclusion Criteria and Study Population

Institutional permission was obtained for a retrospective evaluation of clinical and imaging data. Patients were selected for inclusion in this analysis via a retrospective review of referrals to the multidisciplinary team meeting (MDTM) at our institution over a 10-year period from 1 January 2009 to 1 January 2019. The patients were initially identified by a search of electronic MDTM records for the terms “gliomatosis” and “gliomatosis cerebri”. Then, 10% of the remaining records were manually searched to ensure that no additional patients had been excluded by the initial search strategy. The patients were then excluded if their final diagnosis was not glioma, or if their tumour did not have a gliomatosis growth pattern involving at least three brain lobes on radiology review.

### 2.2. Demographic Data, Imaging and Pathology

Data were collected from the electronic medical record, including age, sex, presenting symptoms and survival. Neuropathology reports were obtained from the electronic medical record, or from clinical correspondence in patients who were referred with a tissue diagnosis determined elsewhere. The results of genetic testing on individual biopsy samples were recorded, including IDH genotype, presence of 1p/19q co-deletion, histone H3F3 K27M mutation and MGMT methylation status.

### 2.3. Imaging Analysis

The imaging studies were reviewed by a qualified neuroradiologist (DD) using Carestream Vue software (Carestream Vue, version 12.1.5.7014, Carestream Health, Inc., Rochester, New York, NY, USA). The patient’s first MRI was interrogated to provide the number of supratentorial lobes and number of total brain regions infiltrated by tumour. In addition to the frontal, parietal, temporal and occipital lobes, other anatomical regions evaluated comprised the brainstem, deep grey nuclei on both sides, corpus callosum and cerebellum. T2/FLAIR mismatch, a predictor of IDH mutant 1p/19q non-co-deleted glioma status, was specified when the tumour was hyperintense signal on T2-weighted sequence but hypointense on fluid-attenuated inversion recovery sequence (FLAIR) [6]. The minimum and mean ADC (ADC_min_ and ADC_mean_) values of the tumour were measured by drawing regions of interest within the tumour volume, and the relative ADC (rADC_mean_) was calculated by comparing tumour ADC_mean_ to a region of normal white matter [7]. All patients had pre- and post-contrast T1-weighted (T1CE) sequences available for review, and the pattern of any contrast enhancement was recorded.

Advanced MRI results (dynamic susceptibility contrast-enhanced (DSC) perfusion and spectroscopy (MRS)) were reviewed by three neuroradiologists (DD, ST, RJ) in addition to the available imaging report. DSC perfusion imaging was analysed using Olea Sphere v2.3 (Olea Medical). A threshold-relative cerebral blood volume (rCBV) value of 1.75 compared to centrum semiovale normal-appearing white matter (NAWM) was adopted to signify elevated perfusion [8]. MRS was performed using chemical shift imaging (echo time 30 to 135 ms).

### 2.4. Statistical Analysis

The data were analysed using GraphPad Prism8 (GraphPad Software, version 8, San Diego, California, CA, USA), a proprietary statistical software package. When calculating the descriptive statistics, the mean values and standard deviation were calculated for normally distributed data; median and interquartile range were calculated for non-normally distributed data. In a Kaplan–Meier survival analysis, patients without a confirmed date of death were censored at the time of their last known contact with our hospital.

## 3. Results

### 3.1. Patient Characteristics and Presenting Symptoms

A total of 29 patients were included in the analysis, of whom 20 were male. The median age at the point of referral to MDT was 64 years old (interquartile range (IQR) 48 to 69 years). 

Many patients had multiple symptoms, but the most common were behavioural or mood disturbance in nine patients (31%), followed by seizures (n = 7, 24%), headache (n = 6, 21%) and focal motor weakness (n = 5, 17%). 

The baseline patient characteristics and presenting symptoms are summarised in Table 1.

### 3.2. Imaging Tumour Characteristics

**Figure 1:**
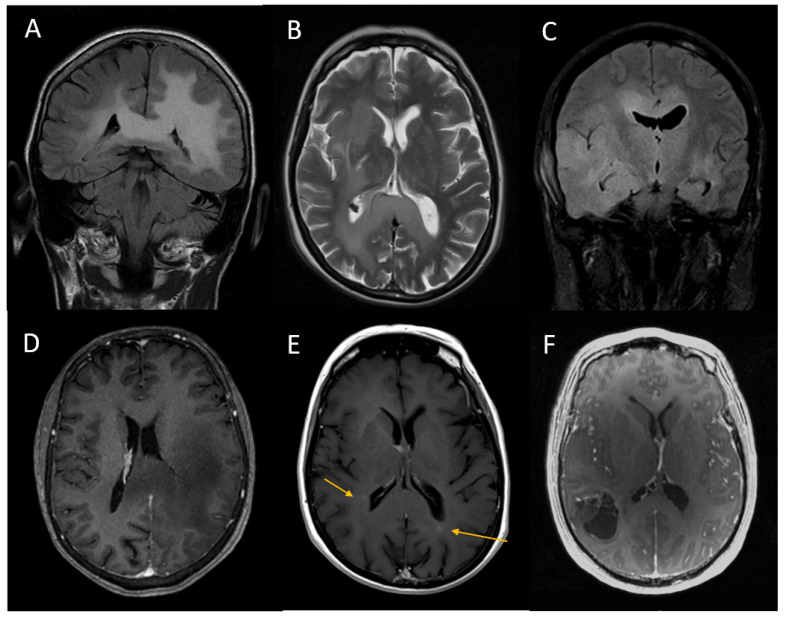
Fluid-attenuated inversion recovery (FLAIR, (**A**,**C**)), T2-weighted (**B**) and gadolinium-enhanced T1-weighted images (**D**–**F**) in three individuals with gliomatosis demonstrating non-enhancing (**D**), patchy solid enhancing ((**E**), arrows) and rim-enhancing (**F**) infiltration.

The median number of supratentorial lobes infiltrated by tumour was 5 (IQR 3 to 8 lobes). The median number of brain regions involved (as defined above) was 7 (IQR 5 to 9 regions). None of the tumours in this study demonstrated T2/FLAIR mismatch. In addition, 64% (16/25) of gliomas for which a T1CE sequence was available demonstrated either patchy solid enhancement (n = 8) or peripheral rim enhancement (n = 4). Examples of these imaging findings are shown in Figure 1. 

The median ADC_mean_ value within tumour was 0.97 × 10^−3^ mm^2^/s (IQR 0.87 to 1.10), while the median ADC of normal white matter elsewhere was 0.75 × 10^−3^ mm^2^/s (IQR 0.73 to 0.82). The ADC_min_ value within each tumour was also measured, with a median of 0.81 × 10^−3^ mm^2^/s (0.64 to 0.90 mm^2^/s). The median rADC_mean_ was 1.29 (IQR 1.20 to 1.34). Imaging features are summarized in Table 2.

**Figure 2:**
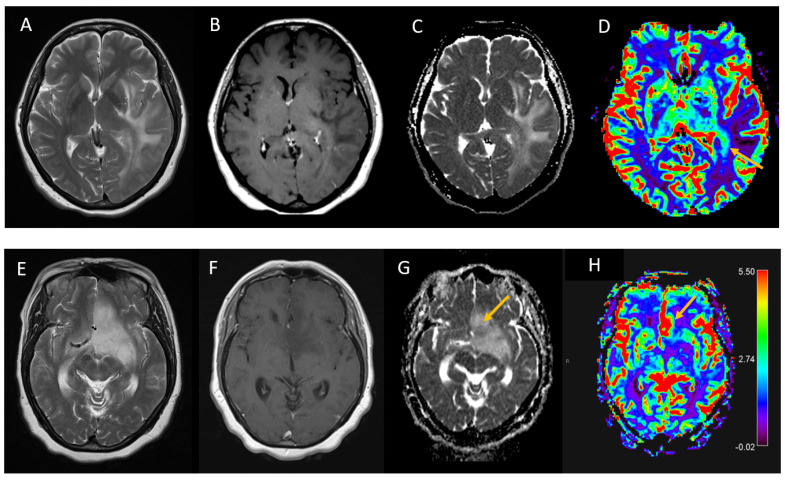
T2-weighted and gadolinium-enhanced T1-weighted images, ADC maps and dynamic susceptibility contrast-enhanced (DSC) imaging-derived rCBV maps in two individuals with WHO grade 4 IDH wild-type glioblastoma (**A**–**D**) and WHO grade 3 IDH1 mutant diffuse astrocytoma (**E**–**H**) showing elevated tumour perfusion (arrows).

Physiological imaging sequences were obtained in eight patients (n = 8 DSC and n = 3 MRS). In the eight patients who had DSC perfusion studies, five (63%) had a measurable region of increased tumour perfusion with rCBV values ranging from 2.8 to 5.7. An analysis of the perfusion imaging at two timepoints >3 months apart by the same observer (ST) and performed independently by one additional observer (RJ) blinded to the initial results showed agreement regarding the presence and site of pathological perfusion in all cases. Typically, the rCBV elevation only involved a small region within the gliomatosis. In only one of three patients who underwent multivoxel MRS were the results considered suggestive of tumour, with a reversal of the normal choline:creatinine ratio (1.17:1), although in the other two patients the spectroscopy data could not be reanalysed as the scans were performed at other institutions. Examples of the diffusion-weighted, DSC and MRS imaging are provided in Figure 2.

### 3.3. Histological Grade and Genetic Tumour Characteristics

Detailed neuropathology reports following biopsy were available for 25/29 patients. Tumour grading was recorded as WHO grade 4 in 8/25 (32%), WHO grade 3 in 6/25 (24%) and WHO grade 2 in 8/25 (32%). In 2/25 (8%) patients, no tumour cells were identifiable in the biopsy specimen, but the serial imaging findings remained consistent with gliomatosis cerebri and no alternative diagnosis could be established. 

Where a definitive histological diagnosis of tumour type was available (n = 22), the commonest tumours were WHO grade 4 glioblastoma (n = 8/22, 36%), WHO grade 2 diffuse astrocytoma (n = 7/22, 32%) and WHO grade 3 diffuse astrocytoma (n = 4/22, 18%). 

Twenty patients had molecular data on tumour IDH mutation status: 15/20 tumours were IDH wild-type and 5/20 (25%) were IDH mutant. All of these were the most common IDH-1 mutation. MGMT methylation status was available in 11 patients, of whom 3 showed methylation in tumour biopsy samples (27%). There were no patients with loss of 1p/19q heterozygosity or BRAF V600E mutation. One patient had a H3 K27M histone-altered tumour. The median Ki67 proliferative index was 10% (IQR 4% to 18%).

Two patients had imaging consistent with gliomatosis cerebri, but the biopsy was non-diagnostic. 

Table 3 lists the final histological diagnosis and WHO grade (where available) for each patient, along with the extent of brain involvement.

### 3.4. Treatment

Specific information about treatment was available for 19/29 patients, of whom 12 had chemotherapy at any time during their treatment (63%) and 9 had radiotherapy (47%). First-line chemotherapy consisted of temozolomide in 11/12 patients, and PCV (procarbazine, CCNU/lomustine and vincristine) in 1/12. Two patients received lomustine as a second-line agent, and one received PCV. Radiotherapy doses varied between 30 Gy in six fractions over 2 weeks and 60 Gy in 30 fractions over 6 weeks. One patient underwent primary radiotherapy (54 Gy in 30 daily fractions) after biopsy in 2010, with a second palliative course of radiotherapy (20 Gy in 5 daily fractions) delivered in 2017. 

### 3.5. Survival

**Figure 3:**
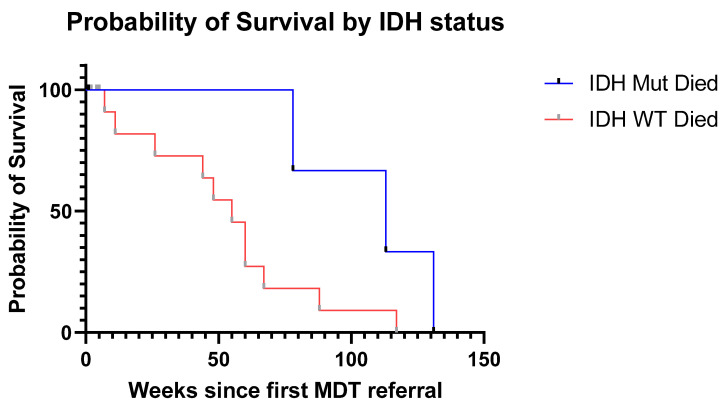
Survival analysis. Kaplan–Meier estimate of length of survival of individual patients in weeks from the date of their first MDT referral. The patients are grouped by IDH status, where known (n = 20). Where no date of death was available, the data were censored at the last known time of contact with our service.

In patients who were known to have died at the time of analysis (n = 21), the median length of survival from MDT referral to death was 48 weeks (IQR 23 weeks to 70 weeks). The Kaplan–Meier survival curve is plotted in Figure 3, stratified by IDH mutation status where known.

## 4. Discussion

Our analysis revealed 29 individuals with gliomatosis cerebri at our specialist centre in a 10-year timeframe. The median length of survival of patients who died was under one year, similar to earlier published case series of patients with gliomatosis [9], and was worse in patients with IDH wild-type tumours.

Although some patients reported “red flag” symptoms at the time of diagnosis [10], including seizures or motor weakness, the non-specific nature of other common complaints such as headache, nausea or behavioural change reinforces the challenge of identifying glioma patients on the basis of their clinical presentation. The presence of neuropsychiatric symptoms such as behavioural change possibly reflects the frequent involvement of the frontal lobe white matter in our patient group [11], although by definition, multiple lobes are involved in each patient.

The defining imaging finding of gliomatosis is multilobar, variably expansile T2/FLAIR signal hyperintensity, with or without enhancement; however, these findings are not unique to tumour and have a differential diagnosis that includes infective or inflammatory disorders. The fact that, in our patient group, 50% of tumours demonstrated contrast enhancement is similar to one other small published cohort [12], but the reported proportion of enhancing tumours varies widely in other research [13,14]. Serial anatomical assessment with repeat MRI did not add value in our experience and risks a diagnostic delay. The role of advanced MRI in diagnosing gliomatosis has been debated; a recent systematic review reported consistent MRS findings indicative of tumour in most (>90 %) gliomatosis patients [15]. Nevertheless, using a state-of-the-art MRS technique (CSI), two thirds (67%) of the results were false-negative in our patient group. Conversely, using DSC perfusion, the finding of at least one region of unequivocally raised rCBV was present in 63% of our cases. This finding is similar to a recently published study that reports a similar frequency of elevated cerebral blood volume in gliomatosis with potential benefit for surgical targeting [16] in a disease that can be fraught with false-negative tissue sampling. For glioma patients, who lack “high grade” anatomical imaging features, the use of perfusion MRI preceding tissue diagnosis is supported by recent international recommendations from the European Society of Neuroradiology [17]. It should be noted that in many gliomatosis patients, there may not be frank neovascularization, necrosis or an increased Ki67 proliferative index [18]. There are currently no study data assessing a direct correlation between gliomatosis focal MR perfusion abnormalities and tissue results. 

From experience, tumour cell density can be relatively low in gliomatosis biopsy specimens. This can affect diagnosis, grading, and limit the use of advanced genetic testing. Our institution’s multidisciplinary team therefore increasingly favours open biopsy where feasible over needle biopsy. The larger sample volume is obtained with the purpose of minimising the rate of negative biopsy and avoid sampling errors in grading. Where this is not possible, we will particularly aim to include advanced imaging to select a biopsy target.

Targeted biopsy to achieve the most accurate histological grade and genetic diagnosis is generally important for glioma therapy, although recent studies have yielded conflicting results when considering the most effective treatment for gliomatosis. A recent publication interrogating the US National Cancer Database found improved survival in patients undergoing chemotherapy compared with patients with receiving radiation therapy or patients in whom surgery achieved near-gross or gross total resection [4]. Their more complete follow-up data demonstrated a median overall survival of approximately 15 months. However, the type of treatment had no overall effect in a cohort similar to ours in Padua, Italy [19].

The WHO tumour grade in our cohort was variable, which could be influenced by sampling limitations, particularly for patients with deep lesions or those without a specific surgical target. IDH1 and MGMT promoter methylation were the two most common mutations in genetic analysis, but these were absent in the majority of patients tested, which is consistent with published genotyping data [15]. 

Gliomatosis cerebri is genetically and epigenetically heterogeneous, which resulted in its removal as a separate entity from the WHO classification [20]. Why this specific growth pattern occurs in individual tumours remains unclear. The discrepancy that two patients had serial clinical and imaging features indicative of gliomatosis, but non-diagnostic biopsy results (despite sufficient sampling), highlights a diagnostic dilemma, which requires close interspecialty communication to optimise therapeutic strategy. 

## 5. Limitations

The identification of the study cohort through our institution’s multidisciplinary team meeting was considered the best available route for capturing all referrals for gliomatosis cerebri. However, it is possible that additional patients in geographically nearby external institutions may not have been referred to the MDT, for example, because of comorbid illness. Likewise, some patients could have bypassed the MDT by immediately opting for conservative management without a tissue diagnosis. The long-term follow-up data were incomplete, particularly in patients living beyond the hospital catchment area. Genetic analysis was not available for all patients, but became increasingly relevant during the study period with the publication of the WHO 2016 Classification of Tumours of the Central Nervous System. The small number of individuals who underwent advanced imaging limits the generalisability of interpreting the diagnostic contributions from MR perfusion or spectroscopy. 

## 6. Conclusions

Gliomatosis imaging features and histological and genetic characteristics are heterogeneous. The prognosis of patients with this tumour growth pattern remains adverse, with a risk of diagnostic delay at the imaging stage, or even following biopsy. In this observational cohort, interval imaging made no positive contribution to the suspected diagnosis. Advanced imaging (perfusion, MRS) could be valuable for targeting surgical biopsy, but depending on the underlying glioma entity, its sensitivity is probably limited. A negative biopsy result should prompt the consideration of alternative diagnoses, but does not necessarily exclude the possibility of gliomatosis. 

## Figures and Tables

**Table 1:** Patient baseline characteristics at the time of first referral to the MDTM.

Patient Characteristic	
Female sex (n, %)	9 (31%)
Age at date of referral (median, IQR)	64 years (48 to 69)
External referral (n, %)	18 (62%)
**Initial symptoms (n, %)**	
- Neuropsychiatric	9 (31%)
- Seizure	7 (24%)
- Headache	6 (21%)
- Focal weakness	5 (17%)
- Language disorder	3 (10%)
- Nausea and vomiting	2 (7%)
- Visual disturbance	2 (7%)

**Table 2:** Imaging features on the first available MRI examination.

Imaging Feature		
Number of supratentorial lobes involved on MRI (mean, range)	5	(3 to 8)
Number of brain regions involved on MRI (mean, range)	7	(5 to 9)
Median lowest ADC_mean_ within the tumour (×10^−3^ mm^2^/s, IQR)	0.81	0.64 to 0.90
Median ADC_mean_ within the tumour (×10^−3^ mm^2^/s, SD)	0.97	0.87 to 1.01
Median ADC_mean_ in normal white matter (×10^−3^ mm^2^/s, SD)	0.75	0.73 to 0.82
T2/FLAIR Mismatch (n, %)	0	(0%)
Advanced imaging performed (DSC perfusion or MRS) (n, %)	8	(28%)

**Table 3:** Tumour WHO grade, histological diagnosis, IDH mutation and number of supratentorial lobes involved on imaging for individual gliomatosis patients.

Patient Number	Age at Referral	WHO Grade (Where Available)	Histological Diagnosis	IDH Mutation Status (Where Available) (1 = Mutant, 0 = Wild Type)	Number of Supratentorial Lobes Involved on Imaging
1	49	2	Diffuse Astrocytoma	1	8
2	67		Non-diagnostic biopsy		3
3	68	4	Glioblastoma	0	8
4	56				6
5	67	4	Glioblastoma	0	8
6	65	3	“Gliomatosis Cerebri”		8
7	51	2	Diffuse Astrocytoma	0	4
8	43				3
9	69	2	Diffuse Astrocytoma	0	8
10	27	2	Diffuse Astrocytoma	0	3
11	64	2	Diffuse Astrocytoma	1	6
12	33	2	Diffuse Astrocytoma	0	6
13	74		“Low Grade Glioma”		2
14	51	4	Glioblastoma	0	4
15	27	3	Anaplastic Astrocytoma	0	4
16	48	2	Diffuse Astrocytoma	1	5
17	80				4
18	70	4	Glioblastoma	0	8
19	68		Non-diagnostic biopsy		5
20	47	3	Anaplastic Astrocytoma	1	5
21	70		Gemistocystic Astrocytoma	0	3
22	43	2	Astrocytoma	1	8
23	18	3	Anaplastic Astrocytoma		3
24	79	4	Glioblastoma	0	3
25	57	3	Pleomorphic Xanthastrocytoma	0	3
26	65	3	Anaplastic Astrocytoma	0	4
27	75	4	Glioblastoma		6
28	71	4	Glioblastoma	0	8
29	54	4	Glioblastoma	0	4

## Data Availability

Extracts of the data presented in this study are available on request from the corresponding author. Data have not been made publicly available due to the rare nature of the diagnosis.
